# Supplementary material for: Differences in topological progression profile among neurodegenerative diseases from imaging data
Source: eLife. 2019 Dec 13;8:e49298. doi: 10.7554/eLife.49298 (PMC6922631; doi:10.7554/eLife.49298)
Supplement: Supplementary file 1. [file elife-49298-supp1.docx]

Supplementary Information

# Topological profiles for 82 regions

In Table S1 we report the topological profiles estimated on the set of 82 FreeSurfer-regions.

| Topological hypotheses | Network metrics | AD | PPMS | HA |
| --- | --- | --- | --- | --- |
|  | Betweenness centrality | 0.24(0.22) | 0.09(0.11) | 0.10(0.07) |
| Centrality | Closeness centrality | 0.01(0.04) | 0.08(0.10) | 0.01(0.02) |
|  | Weighted degree | 0.03(0.04) | 0.09(0.04) | 0.10(0.07) |
|  | Clustering coefficient | 0.17(0.15) | 0.12(0.06) | 0.09(0.09) |
| Segregation | Inverse degree | 0.03(0.07) | 0.21(0.18) | 0.01(0.01) |
|  | Inverse clustering | 0.06(0.03) | 0.34(0.21) | 0.01(0.01) |
| Network proximity | Shortest path | 0.58(0.44) | 0.04(0.05) | 0.04(0.04) |
| Spatial proximity | Spatial distance | 0.02(0.04) | 0.20(0.16) | 0.49(0.37) |
| Constant progression | Constant term | 0.06(0.06) | 0.08(0.06) | 0.14(0.11) |

Table S1. Weights of the topological profiles of the three cohorts when using the entire set of 82 ROIs. The table reports the weights for each network metric, grouped by topological descriptor. Credible intervals for the weights are given in parentheses.

# Topological profiles for age-matched HA subsets

In Table S2 we report the topological profiles for the HA cohort, as well as topological profiles for HA subsets age-matched with AD (age: 73.9±7.2; 1859 subjects) and PPMS (41.6±10.2; 651 subjects).

| Topological hypotheses | Network metrics | HA | HA matched with AD | HA matched with PPMS |
| --- | --- | --- | --- | --- |
|  | Betweenness centrality | 0.09(0.06) | 0.10(0.08) | 0.13(0.12) |
| Centrality | Closeness centrality | 0.03(0.05) | 0.01(0.02) | 0.03(0.04) |
|  | Weighted degree | 0.13(0.09) | 0.12(0.07) | 0.14(0.10) |
|  | Clustering coefficient | 0.10(0.07) | 0.11(0.08) | 0.12(0.11) |
| Segregation | Inverse degree | 0.01(0.01) | 0.01(0.01) | 0.01(0.02) |
|  | Inverse clustering | 0.01(0.01) | 0.01(0.04) | 0.02(0.03) |
| Network proximity | Shortest path | 0.01(0.04) | 0.01(0.02) | 0.01(0.01) |
| Spatial proximity | Spatial distance | 0.64(0.38) | 0.60(0.31) | 0.50(0.39) |
| Constant progression | Constant term | 0.19(0.09) | 0.22(0.10) | 0.25(0.22) |

Table S2. Weights of the topological profiles for the HA cohort, and two subsets age-matched with AD and PPMS. The table reports the weights for each network metric, grouped by topological descriptor. Credible intervals for the weights are given in parentheses.

# Late-stage atrophy modeling

We retrieve the cohort-level late-stage atrophy patterns from the T1-MRI images, taken, for each individual, at their last time-point. We compute the volumes and the z-scores of each of the 41 regions following the pre-processing steps described in Methods: Model inputs pre-processing. Specifically, the z-scores are computed between two distinct individuals groups: (i) individuals diagnosed with “Dementia” at the last time-points and individual diagnosed “Cognitively normal” at the last time-point for the AD cohort; (ii) PPMS-diagnosed individuals and healthy controls, at the last time-point, for the PPMS cohort; (iii) the “Young” and “Old” populations, where each individual is taken at their last time point, for the HA cohort, where “Young” and “Old” are defined in “Supplementary Information on Data”. Atrophy for each region was then normalized between 0 and 1, and was used as the output ***Y*** of model in Eq (4), to obtain a set of descriptors for late-stage data.

# Cluster separation between cohorts

To show that the separation of the three cohorts is not random and driven by the underlying structural connectivity, we compared it to the separation for a model in which the network metrics are computed over random networks. The networks are computed as follows: from the average structural connectome, we generated 100 randomized networks by randomly rewiring the connectome while preserving the total number of connections. We measured the Davis-Bouldin (DB) index for evaluating cluster separation - a function of the ratio of the within-cluster scatter to the between-scatter separation; in this sense, the lower the DB index, the strongest the cluster separation. Table S3 shows DB indices for the topological profiles clusters obtained when using the GP Progression Model for patients (AD+MCI; PPMS; or HA) and the associated random model – see Figure 3a) main text; for the topological profiles clusters obtained when using the GP Progression Model for healthy controls only – see Figure 3c) main text; and for the topological profiles clusters when using end-stage data – see Figure 3e) main text.

| Topological profiles (patients + DPM) | Network model (patients) | Topological profiles (healthy controls + DPM) | Topological profiles (patients +end-stage) |
| --- | --- | --- | --- |
| 0.64 | 0.97 | 0.87 | 0.79 |

Table S3. DB indices for cluster separation induced by the topological profiles when using patients or healthy controls data, and the associated random models. For the random model, the DB index is the averages over the 100 randomized networks.

# Aikake Information Criteria for model selection: topological profiles against best-fitting descriptor

|  | AD | PPMS | HA |
| --- | --- | --- | --- |
| Topological profile model | 24.4 | 24.8 | 20.1 |
| Single-best fitting descriptor | 35.9 | 31.0 | 27.6 |

Table S4. AIC scores for the topological profile model and the single-best fitting descriptor model, computed for each cohort (AD, PPMS and HA).

# ANOVA results for clinical and demographic comparison

|  | outliers (number) | diagnosis  (p-values) | age  (p-values) | gender  (p-values) | YoE  (p-values) | APOE4  (p-values) | AV45  (p-values) | MMSE  (p-values) | ADAS13  (p-values) | CDRSB  (p-values) |
| --- | --- | --- | --- | --- | --- | --- | --- | --- | --- | --- |
| AD in HA | 159 | 0.74 | 0.11 | 0.60 | 0.10 | 0.23 | 0.88 | **0.01** | 0.08 | 0.06 |
| AD in PPMS | 53 | 0.74 | 0.19 | 0.09 | 0.22 | 0.13 | 0.09 | 0.24 | 0.74 | 0.72 |

Table S5a). Full information on comparisons between groups (outliers vs not-outliers) in terms of p-values of the ANOVA test, when outliers are defined by using the DPM model, for the AD cohort. Significant results in bold. YoE: years of education; MMSE: mini mental state examination; AV45: (18)F-florbetapir Amyloid PET imaging; ADAS13: Alzheimer’s Disease Assessment Scale-cognitive subscale, 13 items; CDRSB: clinical diagnosis rating scale – sum of boxes.

|  | Outliers (number) | age  (p-values) | gender  (p-values) | duration  (p-values) | EDSS  (p-values) |
| --- | --- | --- | --- | --- | --- |
| PPMS in AD | 1 | 0.31 | 0.37 | 0.74 | 0.82 |
| PPMS in HA | 13 | 0.09 | 0.21 | 0.50 | 0.26 |

Table S5b). Full information on comparisons between groups (outliers vs not-outliers) in terms of p-values of the ANOVA test, when outliers are defined by using the DPM model, for the PPMS cohort. EDSS: expanded disability status scale.

|  | Outliers (number) | age  (p-values) | Gender  (p-values) | APOE4  (p-values) | MMSE  (p-values) |
| --- | --- | --- | --- | --- | --- |
| HA in AD | 358 | **0.05** | 0.70 | 0.41 | **<0.01** |
| HA in PPMS | 100 | 0.56 | 0.11 | 0.67 | 0.44 |

Table S5c). Full information on comparisons between groups (outliers vs not-outliers) in terms of p-values of the ANOVA test, when outliers are defined by using the DPM model, for the HA cohort. Significant results in bold. MMSE: mini mental state examination.

|  | outliers (number) | diagnosis  (p-values) | age  (p-values) | gender  (p-values) | YoE  (p-values) | APOE4  (p-values) | AV45  (p-values) | MMSE  (p-values) | ADAS13  (p-values) | CDRSB  (p-values) |
| --- | --- | --- | --- | --- | --- | --- | --- | --- | --- | --- |
| AD in HA | 441 | 0.98 | 0.29 | 0.10 | 0.87 | 0.31 | 0.40 | 0.19 | 0.08 | 0.63 |
| AD in PPMS | 122 | 0.25 | 0.27 | 0.45 | 0.20 | 0.18 | 0.29 | 0.96 | 0.59 | 0.98 |

Table S6a). Full information on comparisons between groups (outliers vs not-outliers) in terms of p-values of the ANOVA test, when outliers are defined by using late-stage data only, for the AD cohort. YoE: years of education; MMSE: mini mental state examination; AV45: (18)F-florbetapir Amyloid PET imaging; ADAS13: Alzheimer’s Disease Assessment Scale-cognitive subscale, 13 items; CDRSB: clinical diagnosis rating scale – sum of boxes.

|  | Outliers (number) | age  (p-values) | gender  (p-values) | duration  (p-values) | EDSS  (p-values) |
| --- | --- | --- | --- | --- | --- |
| PPMS in AD | 7 | 0.34 | 0.88 | 0.97 | 0.34 |
| PPMS in HA | 17 | 0.29 | 0.87 | 0.16 | 0.29 |

Table S6b). Full information on comparisons between groups (outliers vs not-outliers) in terms of p-values of the ANOVA test, when outliers are defined by using late-stage data only, for the PPMS cohort. EDSS: expanded disability status scale.

|  | Outliers (number) | age  (p-values) | Gender  (p-values) | APOE4  (p-values) | MMSE  (p-values) |
| --- | --- | --- | --- | --- | --- |
| HA in AD | 775 | **0.05** | 0.82 | 0.34 | 0.07 |
| HA in PPMS | 631 | 0.56 | 0.12 | 0.62 | 0.43 |

Table S6c). Full information on comparisons between groups (outliers vs not-outliers) in terms of p-values of the ANOVA test, when outliers are defined by using late-stage data only, for the HA cohort. MMSE: mini mental state examination.

# Supplementary Information on Data

Here we report full information on longitudinal data (Figure S5) and on their socio-demographic, for the three data sets: AD (Table S7), PPMS (Table S8) and HA (Table S9).

| socio-demo | HC | MCI | AD |
| --- | --- | --- | --- |
| n (females) | 401(206) | 974(418) | 338(151) |
| age (std) | 74.9(5.8) | 73.2(7.6) | 75.0(7.8) |
| MMSE (std) | 29.1(1.1) | 27.6(1.8) | 23.2(2.1) |
| YoE (std) | 16.3(2.7) | 15.9(2.8) | 15.2(3.0) |
| ADAS13 (std) | 9.3(4.3) | 16.5(6.8) | 29.9(8.1) |
| CDRSB (std) | 1.6(1.7) | 1.4(0.9) | 4.4(1.7) |
| APOE+ (-) | 114(300) | 12(21) | 224(112) |
| AV45 (std) | 1.1(0.2) | 1.2(0.2) | 1.4(0.2) |
| FDG (std) | 1.3(0.1) | 1.2(0.1) | 1.1(0.1) |

Table S7. Baseline sociodemographic and clinical information for the AD study cohort (1713 individuals). HC: healthy controls, MCI: mild cognitive impairment, AD: Alzheimer’s patients. MMSE: Mini Mental State Examination; YoE: years of education; ADAS13: Alzheimer’s Disease Assessment Scale-cognitive subscale, 13 items; CDRSB: Clinical Dementia Rating Scale; AV45: (18)F-florbetapir Amyloid PET imaging; FDG: (18)F-fluorodeoxyglucose PET imaging.

| socio-demo | all | HC | PPMS |
| --- | --- | --- | --- |
| n (females) | 64 | 20(8) | 44(24) |
| age (std) | 41.6(10.2) | 35.1(5.7) | 44.7(10.5) |
| EDSS (std) | 3.1(2.5) | 0(0) | 4.7(1.4) |
| duration (std) | 2.2(1.7) | 0(0) | 3.3(0.9) |

Table S8. Baseline sociodemographic and clinical information for the PPMS study cohort (64 individuals). HC: healthy controls, PPMS: primary progressive patients. EDSS: Expanded Disability Status Scale.

| socio-demo | all | Young | Old |
| --- | --- | --- | --- |
| n (females) | 5463(3036) | 972(535) | 1076(631) |
| age (std) | 64.8(10.8) | 50.5(2.1) | 81.1(4.2) |
| MMSE (std) | 28.0(1.8) | 28.2(1.7) | 27.4(2.1) |
| APOE+ (-) | 1476(3740) | 264(650) | 247(781) |
| dementia developers | 148 | 1 | 93 |
| probable AD | 100 | 0 | 65 |
| AD + VaD | 16 | 0 | 10 |
| VaD | 5 | 0 | 3 |
| PD | 1 | 0 | 0 |
| Other | 26 | 1 | 15 |

Table S9. Baseline sociodemographic and clinical information for the HA study cohort (5463 individuals). Individuals are separated in 2 sub-cohorts: Young and Old, where Young are individuals whose age is below one standard deviation the mean age of the whole cohort, and Old is above 1 standard deviation of the mean age of the whole cohort. Dementia developers refers to the individuals that, healthy at BL, developed dementia in 2-4 years. Lines below are specific to the type of dementia developed; VaD stands for vascular dementia, PD for Parkinson’s disease, and “Other” for other types of dementia, either diagnosed or undetermined.
